# Protective role of vascular endothelial growth factor in endotoxin-induced acute lung injury in mice

**DOI:** 10.1186/1465-9921-8-60

**Published:** 2007-08-25

**Authors:** Hidefumi Koh, Sadatomo Tasaka, Naoki Hasegawa, Wakako Yamada, Mie Shimizu, Morio Nakamura, Makoto Yonemaru, Eiji Ikeda, Yoshiyuki Adachi, Seitaro Fujishima, Kazuhiro Yamaguchi, Akitoshi Ishizaka

**Affiliations:** 1 Division of Pulmonary Medicine, Keio University School of Medicine, Tokyo, Japan; 2 Department of Pathology, Keio University School of Medicine, Tokyo, Japan; 3 Laboratory of Immunopharmacology of Microbial Products, Tokyo University of Pharmacy and Life Science, Tokyo, Japan; 4 Department of Emergency and Critical Care Medicine, Keio University School of Medicine, Tokyo, Japan

## Abstract

**Background:**

Vascular endothelial growth factor (VEGF), a substance that stimulates new blood vessel formation, is an important survival factor for endothelial cells. Although overexpressed VEGF in the lung induces pulmonary edema with increased lung vascular permeability, the role of VEGF in the development of acute lung injury remains to be determined.

**Methods:**

To evaluate the role of VEGF in the pathogenesis of acute lung injury, we first evaluated the effects of exogenous VEGF and VEGF blockade using monoclonal antibody on LPS-induced lung injury in mice. Using the lung specimens, we performed TUNEL staining to detect apoptotic cells and immunostaining to evaluate the expression of apoptosis-associated molecules, including caspase-3, Bax, apoptosis inducing factor (AIF), and cytochrome C. As a parameter of endothelial permeability, we measured the albumin transferred across human pulmonary artery endothelial cell (HPAEC) monolayers cultured on porous filters with various concentrations of VEGF. The effect of VEGF on apoptosis HPAECs was also examined by TUNEL staining and active caspase-3 immunoassay.

**Results:**

Exogenous VEGF significantly decreased LPS-induced extravascular albumin leakage and edema formation. Treatment with anti-VEGF antibody significantly enhanced lung edema formation and neutrophil emigration after intratracheal LPS administration, whereas extravascular albumin leakage was not significantly changed by VEGF blockade. In lung pathology, pretreatment with VEGF significantly decreased the numbers of TUNEL positive cells and those with positive immunostaining of the pro-apoptotic molecules examined. VEGF attenuated the increases in the permeability of the HPAEC monolayer and the apoptosis of HPAECs induced by TNF-α and LPS. In addition, VEGF significantly reduced the levels of TNF-α- and LPS-induced active caspase-3 in HPAEC lysates.

**Conclusion:**

These results suggest that VEGF suppresses the apoptosis induced by inflammatory stimuli and functions as a protective factor against acute lung injury.

## Background

Vascular endothelial growth factor (VEGF) was originally discovered as a vascular permeability factor in guinea pig skin, and is a mitogen that regulates endothelial cell differentiation, angiogenesis, and the maintenance of existing vessels [1-4]. VEGF is involved in the pathogenesis of rheumatoid arthritis, diabetic retinopathy, and tumor growth, and may contribute to endothelial cell migration and proliferation [5,6]. VEGF is expressed primarily on alveolar epithelial cells and activated alveolar macrophages [7-9]. In healthy human subjects, VEGF protein levels in oxygenated alveoli are 500 times higher than in plasma, despite the lack of occurrence of angiogenesis, edema or excess microvascular permeability [10]. These data suggest an important persistent or additional function of VEGF within the human lung that has not yet been characterized.

Acute lung injury (ALI) and its more severe form, acute respiratory distress syndrome (ARDS), involve a disruption of the alveolar-capillary membranes, with local inflammation ultimately leading to alveolar flooding with serum proteins and edema fluid [11,12]. Since ALI/ARDS is characterized by permeability edema, it has been hypothesized that VEGF may contribute to the development of ALI/ARDS. Indeed, the overexpression of VEGF by adenovirus in the lung leads to pulmonary edema and increased lung vascular permeability [13]. To date, however, most observational studies of lung injury in humans have shown a reduction in intrapulmonary VEGF levels in ALI/ARDS, especially in its early stages [14-16]. In a recent study using bronchoscopic microsampling method, we observed greater VEGF levels in epithelial lining fluid (ELF) in the ALI/ARDS patients who survived than in those who did not [17]. In addition, VEGF concentration in ELF was inversely correlated with lung injury score [17]. These findings suggest that the higher VEGF levels in the airspace may be associated with a better outcome for patients with ALI/ARDS.

Apoptosis of endothelial and epithelial cells, which is induced by a variety of stimuli, contributes to the impairment of the barrier function of pulmonary endothelium and epithelium and development of pulmonary edema [18]. There have been several reports describing the anti-apoptotic effect of VEGF on endothelial cells [19-21]. We hypothesized that the role of VEGF may be modified in injured lung. To the best of our knowledge, there has been no report examining both endothelial permeability and apoptosis in a single model of lung injury.

To evaluate the role of VEGF in the apoptosis of endothelial cells and their barrier function in the injured lung, we evaluated the effects of exogenous VEGF and VEGF blockade by monoclonal antibody using a murine model of LPS-induced lung injury. Using the lung specimens, TUNEL staining and immunostaining of caspase-3, Bax, apoptosis inducing factor (AIF) and cytochrome C were performed to detect apoptotic cells and the pro-apoptotic molecules expressed. We also determined the in vitro effects of VEGF on endothelial permeability, apoptosis, and caspase-3 activation using cultured human pulmonary artery endothelial cells (HPAEC). To investigate the mechanism underlying this attenuation of endothelial damage, we evaluated the effect of VEGF on apoptosis and the level of active caspase-3, a distal enzyme in the caspase cascade, in endothelial cells.

## Methods

### Reagents

Purified recombinant human TNF-α and recombinant human VEGF_165 _were purchased from Pepro Tech, Inc (Rocky Hill, NJ). LPS and bovine serum albumin were obtained from Sigma Chemical Co. (St. Louis, MO). The Bio-Rad Protein Assay kit was obtained from Bio-Rad (Richmond, CA). Recombinant mouse VEGF (rmVEGF) and anti-mouse VEGF antibody (anti-VEGF Ab) was obtained from R&D systems.

### Murine model of acute lung injury

The experimental protocol was approved by the Keio University Council on Animal Care in accordance with the guidelines of the National Institute of Health. The effect of exogenous VEGF and anti-VEGF antibody was examined in a murine LPS-induced lung injury model as previously described [22]. The experimental protocol was approved by the Keio University Council on Animal Care in accordance with the guidelines of the National Institute of Health. To determine the effect of VEGF on LPS-induced acute lung injury, five groups of C57BL/6 mice (8 weeks old, CLEA Japan, Tokyo, Japan) were studied (n = 6 each). Control group was given an intravenous injection of 100 μl saline 24 and 1 h before intratracheal instillation of PBS (50 μl); LPS group received 100 μl saline intravenously 24 and 1 h before LPS (10 μg/body in 50 μl PBS) challenge; LPS+anti-VEGF group received anti-VEGF antibody (25 μg/body in 100 μl PBS) intravenously 10 min before and 2 h after LPS challenge; LPS+VEGF (pre) was given intravenous injection of rmVEGF (1 μg/body in 100 μl PBS) 24 and 1 h before LPS challenge; LPS+VEGF (post) was given intravenous injection of rmVEGF (1 μg/body in 100 μl PBS) 10 min and 2 h after LPS challenge.

Mice were anesthetized using ketamine hydrochloride (80–100 mg/kg i.m.) and acepromazine maleate (5–10 mg/kg i.m.). Anesthetized mice received an intravenous injection of ^125^I-labeled human albumin (0.1 mCi/mouse) 15 min before intratracheal administration of either PBS or LPS. Two minutes before the end of the study, ^131^I-albuimn (0.1 mCi/mouse) was injected to estimate the intravascular blood volume. The separation rate of the labeled albumin was less than 1%. After 6 h, the lungs were excised by opening the chest, and free blood was removed by blotting the hilus on paper towels.

The gamma counts of tissue samples for ^125^I and ^131^I were determined in a gamma well counter with appropriate corrections for cross over. The tissues were then dried in a vacuum drying oven at 90°C and -200 mmHg for 48 h. The weights of the dried lung tissue samples were determined. Blood contamination in each sample was estimated from ^131^I counts of the tissue sample, and extravascular lung water was determined by calculating the lung tissue wet-to-dry weight (W/D) ratio after correcting the contamination [23]. Transvascular flux of ^125^I-albumin was assessed by control ratio of lung tissue to plasma per unit weight, which was used to estimate vascular endothelial damage (^125^I-albumin lung tissue-plasma ratio: lung T/P ratio).

For lung pathology, the lungs were removed and fixed by intratracheal instillation of 6% glutaraldehyde at 22 cmH_2_O. Paraffin-embedded 5-μm sections of lungs were cut and stained with hematoxylin and eosin. To evaluate lung tissue edema, Interstitial area/Total lung area ratio (I/T ratio) was calculated from the cross-sectional areas of lung interstitium and total lung using Micro Analyzer software (Japan Poladigital, Tokyo, Japan). Slides from each animal were observed under microscope and photographed in 30 randomly selected fields at ×200. The mean I/T ratio of each animal was employed and compared between the experimental groups. In addition, neutrophil emigration was evaluated by counting the number of neutrophils in 200 randomly selected alveoli and was expressed as the number of neutrophils per 100 alveoli. The microscopic observations were conducted by single investigator in a blinded fashion.

### TUNEL staining of the lung section

TUNEL staining was performed with MEBSTAIN II Apoptosis kit (Medical&Biological Laboratories, Nagoya, Japan), following the manufacturer's instructions. After deparaffinization and rehydration, sections were digested with proteinase K for 30 min. After washing with DW, the slides were immersed in buffer. TdT, 1 mM Mn^2+^, and biotinylated dUTP in TdT buffer were then added to cover the sections and incubated at 37°C for 60 min. After washing with DW, endogenous peroxidase activity was quenched with 0.3% H_2_O_2 _for 5 min. The slides were washed with PBS, covered with PBA for 10 min. After rinsing with PBS, the slides were covered with extra-avidin peroxidase (Dako Japan, Kyoto, Japan) and immersed in DAB solution. The slides were counterstained for 20 sec with Mayer-hematoxylin, dehydrated, and mounted.

### Immunohistochemistry of the lung section

Anti-human caspase-3 rabbit polyclonal antibody (Novocastra Laboratories, Newcastle, UK), anti-human Bax rabbit polyclonal antibody (Oncogene, San Diego, CA), anti-human AIF rabbit polyclonal antibody (Biocarta, Carlsbad, CA) and mouse anti-cytochrome C monoclonal antibody (Chemicon International, Temecula, CA) were used. These anti-human antibodies cross-react with mouse antigen.

Lung tissues were fixed in buffered formalin, embedded in paraffin and sectioned (5 μm) for immunohistochemical assays. The sections were treated with 0.3% H_2_O_2 _in methanol for 5 min. The slides were washed with PBS and blocked with 1% PBA for 5 min. Anti-human caspase-3 rabbit polyclonal antibody (1:750 dilution), anti-human Bax rabbit polyclonal antibody (1:100 dilution), anti-human AIF rabbit polyclonal antibody (1:1500 dilution) and mouse anti-cytochrome C monoclonal antibody (1:400 dilution) were incubated with the slides at 4°C for overnight, followed by biotinylated pig anti-Rabbit IgG (1:300 dilution, Dako) or biotinylated secondary antibody (1:400 dilution) and extra-avidin peroxidase for 10 min each. After rinsing with PBS, the slides were immersed in DAB solution for 5 min. The slides were counterstained for 20 sec with Mayer-hematoxylin, dehydrated, and mounted. The slides were evaluated in a blinded fashion.

### Preparation of the endothelial monolayer

Human pulmonary artery endothelial cells (HPAEC) were purchased from KURABO (Osaka, Japan) at 3rd passage. In a humidified 5%CO_2 _atmosphere, the endothelial cells were maintained with a culture medium (Humedia-EB2) supplemented with 2% fetal bovine serum, 10 ng/ml recombinant human EGF, 1 μg/ml hydrocortisone, 50 μg/ml gentamicin, 50 ng/ml amphotericin B, 5 ng/ml recombinant human FGF-β and 10 μg/ml heparin. Only cells from passages four to six were studied in these experiments.

Preparation of the endothelial monolayer has been described in detail previously [24]. Millicell-HA tissue culture plate well inserts (12 mm diameter) were obtained from Millipore (Bedford, MA). The inserts consisted of a surfactant-free 0.45 μm pore size microporous membrane filter (manufactured of mixed cellulose) sealed to a cylindrical polystyrene holder with an effective membrane surface area of 0.6 cm^2^. HPAECs suspended in the culture medium were seeded on the membrane filter at a density of 4 × 10^5 ^cells/filter insert. The inserts were incubated and placed into 6-well culture plates (Corning Laboratory Science, Corning, NY) until permeability measurements were made.

### Measurement of permeability of endothelial monolayer

As a parameter of endothelial permeability, we measured the albumin transferred across the HPAEC monolayers cultured on porous filter [24]. HPAEC were incubated with TNF-α, LPS and VEGF or combination described below. The experimental groups were as following;

TNF-α group: incubated with 100 ng/ml of TNF-α for 24 h, LPS group: incubated with 100 ng/ml of LPS for 24 h, TNF-α+VEGF group: after pre-incubation with 20, 100 and 500 ng/ml VEGF for 48 h, HPAEC were co-incubated with VEGF and 100 ng/ml TNF-α for 24 h, LPS+VEGF group: after pre-incubation with 100 and 500 ng/ml VEGF for 48 h, HPAEC were co-incubated with VEGF and 100 ng/ml LPS for 24 h.

HPAEC monolayers were incubated with culture medium containing test agent for the experimental protocol at 37°C in a humidified 5% CO_2 _atmosphere. After aspiration of culture medium, 500 μl of PBS containing 1 mg/ml bovine serum albumin was added to the upper chamber (the filter insert). The insert was placed in one well of a 24-well culture plate (Falcon, Becton Dickinson, Franklin Lakes, NJ). After incubation for 20 min, the insert was removed from the well. The albumin concentration of the lower chamber was measured with Bio-Rad Protein Assay kit (Bio-Rad, Hercules, CA).

### TUNEL staining of cultured endothelial cells

Terminal deoxynucleotidyl transferase-mediated dUTP nick end-labeling (TUNEL) was performed with Apoptosis in situ Detection kit (Wako Pure Chemical Industries, Osaka, Japan). HPAECs were incubated with culture medium containing test agent for the experimental protocol (similar to permeability study). After aspiration of the test agent and culture medium, HPAEC were fixed with 4% buffered formaldehyde for 10 min and permeabilized with 0.1% Triton X in sodium citrate for 2 min on ice. After washing with PBS, the slides were immersed in terminal deoxynucleotidyl transferase (TdT) buffer and incubated in a humid atmosphere at 37°C for 10 min. After washing with PBS, endogenous peroxidase activity was quenched with 3% H_2_O_2 _for 5 min. The slides were washed with PBS and incubated with POD-conjugated antibody in a humid atmosphere at 37°C for 10 min. After rinsing with PBS, the slides were immersed in DAB solution for 5 min. The slides were counterstained for 5 min with 1% methyl green. Cells were observed under microscope and photographed. Apoptotic cell number was counted in 30 randomly selected fields at ×200 and expressed as the average number of apoptotic cells per field.

### Active caspase-3 immunoassay

The HPAEC suspended in the culture medium were seeded on the six-well culture plate and grown to 90% confluence. The HPAEC were incubated with culture medium containing test agent for the experimental protocol (similar to permeability study). After aspiration of the test agent and culture medium, extraction buffer containing protease inhibitors were added, and HPAEC were scraped. Six-well culture plates were covered and set at room temperature for 2 h. Calibrator diluent was added and we obtained 1 × 10^6 ^cells/ml extract sample.

To measure active caspase-3, we used human active caspase-3 immunoassay (R&D systems, Minneapolis, MN). Standards and cell extract samples containing covalently linked active caspase-3 biotin-ZVKD were pipetted into the wells, and any caspase-3 present was bound by the immobilized antibody. Inactive caspase-3 zymogen is not modified by biotin-ZVKD-fmk inhibitor and therefore was not detected. Following a wash to remove any unbound substances, streptavidin conjugated to horseradish peroxidase (HRP) was added to the wells and binds to the biotin on the inhibitor. Following a wash to remove any unbound streptavidin-HRP reagents, a substrate solution was added to the wells. The intensity of the color measured was in proportion to the amount of active caspase-3 bound in the initial step. The sample values were then read off the standard curve, followed by cell extract samples. The optical density was measured at the wavelength of 450 nm using a microplatereader SJeia II (Sanko Junyaku, Tokyo, Japan) with correction of wavelength of 570 nm.

### Statistical analysis

All values are expressed as mean ± SEM. One-way analysis of variance (ANOVA) and Scheffe test were used to detect differences between groups. Statistical significance was defined as p < 0.05.

## Results

### Pulmonary endothelial permeability

**Figure 1:**
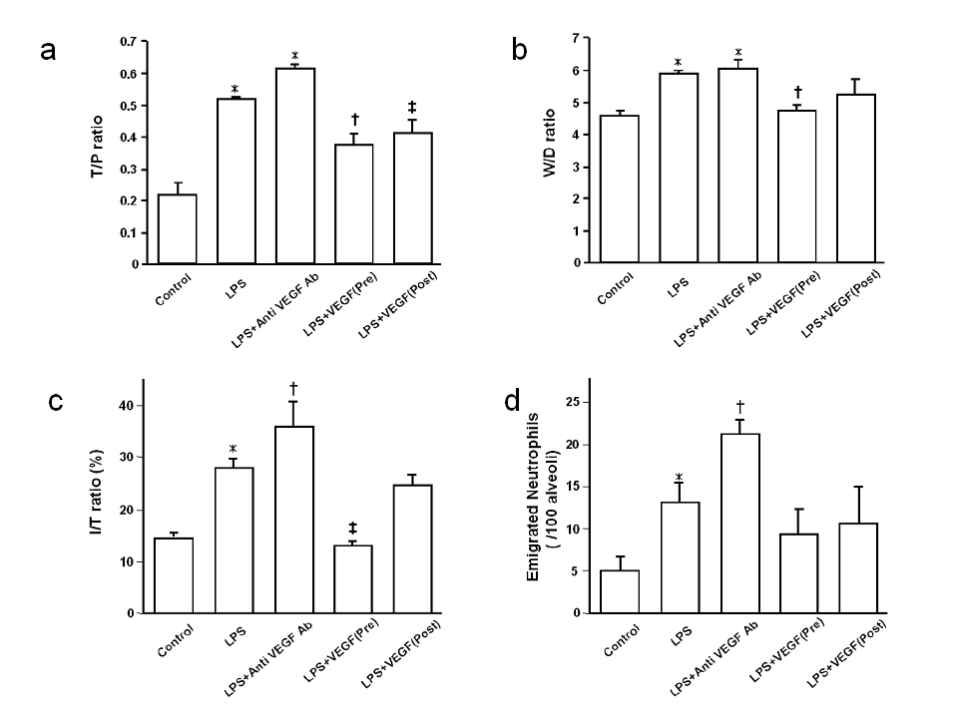
**Effect of exogenous VEGF and anti-VEGF antibody on LPS-induced lung injury**. **a) **T/P ratio 6 h after intratracheal LPS instillation. Mice received treatment with either PBS, VEGF or anti-VEGF Ab intravenously. The group with LPS challenge revealed increased T/P ratio (*p < 0.0001). The T/P ratio of LPS+anti VEGF Ab group tends to increase compared with that of LPS group. In the LPS+VEGF (pre- and post-treatment) groups, T/P ratio was significantly decreased compared with LPS group (^†^p < 0.01, ^‡^p < 0.05, respectively). n = 6 in each group. **b) **W/D ratio 6 h after intratracheal LPS instillation. Mice received treatment with either PBS, VEGF or anti-VEGF Ab intravenously. The group with LPS challenge revealed increased W/D ratio (*p < 0.01). The W/D ratio of LPS+anti VEGF Ab was not significantly different from that of the LPS group. In the LPS+VEGF (Pre) group, W/D ratio was significantly decreased compared with the LPS group (^†^p < 0.05). n = 6 in each group. **c) **I/T ratio 6 h after intratracheal LPS instillation. The group with LPS challenge revealed increased I/T ratio (*p < 0.0001) compared with the control group. In the anti-VEGF Ab group, I/T ratio was greater than LPS group (^†^p < 0.05). In the LPS+VEGF (pre) group, I/T ratio was significantly decreased compared with LPS group (^‡^p < 0.0001). n = 6 in each group. **d) **Emigrated neutrophils 6 h after intratracheal LPS instillation. Mice received treatment with either PBS, VEGF or anti-VEGF Ab intravenously. The LPS group revealed an increase in emigrated neutrophil (*p < 0.05). Treatment with anti VEGF Ab significantly enhanced neutrophil emigration, compared with the LPS group (^†^p < 0.05). Pre- or post-treatment with VEGF did not affect neutrophil emigration, compared with the control and the LPS groups. n = 6 in each group.

We measured the lung T/P ratio to assess albumin leakage into the pulmonary interstitium (Fig. 1a). LPS treatment significantly increased the T/P ratio compared with the control animals (*p *< 0.0001). Anti-VEGF antibody made no difference in T/P ratio compared with the LPS group, suggesting that inhibition of VEGF might not influence LPS-induced pulmonary endothelial damage. Both pre- and post-treatment with rmVEGF significantly inhibited this LPS-induced increase in the T/P ratio (*p *< 0.01, *p *< 0.05, respectively). It was indicated that exogenous VEGF may attenuate the albumin leakage from lung microvasculature induced by intratracheal LPS.

### Pulmonary edema formation

We measured the W/D ratio to evaluate extravascular lung water (Fig. 1b). LPS treatment significantly increased the W/D ratio compared with the control animals (*p *< 0.01). Anti-VEGF antibody made no difference in W/D ratio compared with the LPS group, suggesting that inhibition of VEGF might not affect LPS-induced increase in lung edema formation. Pre-treatment with rmVEGF significantly attenuated the LPS-induced increase in the W/D ratio (*p *< 0.05), whereas no difference was observed between the LPS and the LPS+VEGF (post) groups.

Using a microanalyzer, we determined the I/T ratio of each field for quantitative evaluation of edema formation in lung interstitium. An average I/T ratio was calculated for each animal and shown in Fig. 1c. The I/T ratio in the LPS group was significantly greater than in the control group (*p *< 0.001). The I/T ratio in the LPS+ anti-VEGF antibody group was significantly greater than in the LPS group (*p *< 0.05). The I/T ratio of the LPS+VEGF (pre) group was significantly decreased compared with the LPS group (*p *< 0.0001), whereas the I/T ratio did not differ between the LPS and LPS+VEGF (post) groups. It was revealed that the interstitial changes induced by intratracheal LPS are attenuated by exogenous VEGF and accentuated by the blockade of the VEGF cascade.

### Neutrophil emigration in alveolar spaces

Emigrated neutrophils were quantified morphologically in histologic sections (Fig. 1d). LPS treatment significantly increased the W/D ratio compared with the control animals (*p *< 0.05). Anti-VEGF antibody significantly enhanced neutrophil emigration compared with the LPS group (*p *< 0.05). Emigrated neutrophils in the LPS+VEGF (pre) and the LPS+VEGF (post) groups were not significantly different from those in the LPS group.

### TUNEL staining of lung specimens

**Figure 2:**
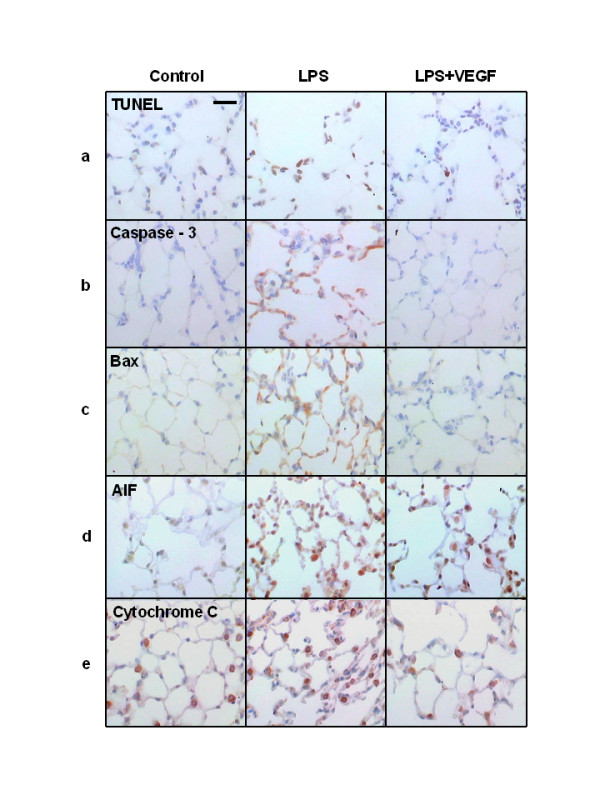
**Representative appearances of lung tissue specimen after TUNEL and immunohistochemical staining**. **a) **In the LPS group, characteristic chromatin condensation in the nuclei of TUNEL-positive epithelial and endothelial cells were observed, which were decreased in the LPS+VEGF group. **b-e) **Caspase-3 **(b)**, Bax **(c)**, AIF **(d) **and cytochrome C **(e) **immunostaining were present in epithelial and endothelial cells, but not in macrophages and neutrophils. In the LPS+VEGF group, TUNEL, caspase-3, Bax, AIF and cytochrome C positive cells were decreased compared with the LPS group. Scale bar = 50 μm.

We performed TUNEL staining on lung sections to determine the effect of VEGF on LPS-induced apoptosis of lung cells (Fig. 2a). We observed characteristic chromatin condensation in the nuclei of TUNEL-positive epithelial and endothelial cells in the LPS group. There were fewer TUNEL-positive cells in the LPS+VEGF group than in the LPS group, suggesting that exogenous VEGF attenuates LPS-induced apoptosis of epithelial and endothelial cells.

### Immunohistochemistry of lung specimens

We performed immunohistochemical staining of lung tissue specimens with the anti-human caspase-3 rabbit polyclonal antibody (Fig. 2b), anti-human Bax rabbit polyclonal antibody (Fig. 2c), anti-human AIF rabbit polyclonal antibody (Fig. 2d), and anti-mouse anti-cytochrome C monoclonal antibody (Fig. 2e). In the LPS group, caspase-3, Bax, AIF, and cytochrome C immunostaining were present in epithelial and endothelial cells, but not in macrophages and neutrophils. VEGF treatment decreased the number of cells positive for these markers, indicating that exogenous VEGF inhibited the apoptosis cascade induced by intratracheal LPS in epithelial and endothelial cells.

### Permeability of endothelial monolayer

To determine the protective effect of VEGF on the endothelial cells, we measured the albumin influx into the lower chamber through the endothelial monolayer. If the tightness of the monolayer is kept, little albumin travels through. Compared with the controls, treatment with TNF-α at concentrations of (100 and 1,000 ng/ml) for 24 h caused a dose-dependent increase in the albumin concentration in the lower chamber (data not shown). TNF-α at 1,000 ng/ml induced a similar increase as 100 ng/ml, suggesting that 100 ng/ml of TNF-α is sufficient to evaluate the effects of VEGF on the endothelial injury induced by TNF-α. On the other hand, VEGF alone (20, 100 and 500 ng/ml) did not change albumin transfer, although the expression of two types of VEGF receptor, VEGF-R1 and VEGF-R2, on HPAEC was confirmed (data not shown).

**Figure 3:**
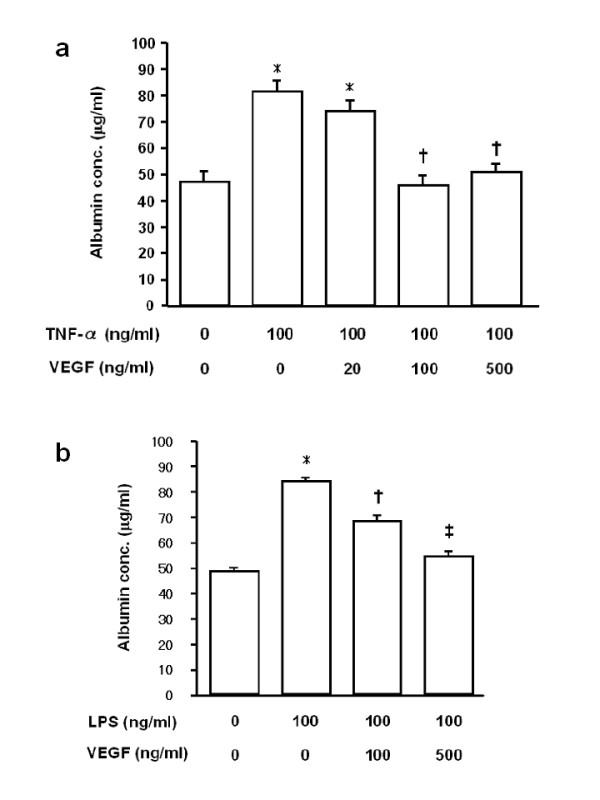
**Effect of VEGF on endothelial damage induced by TNF-α and LPS**. **a) **VEGF significantly reduced albumin transfer to the lower chamber that was induced by TNF-α stimulation for 24 h. *p < 0.001 vs control; †p < 0.001 vs TNF-α. **b) **VEGF significantly inhibited LPS-induced albumin transfer to the lower chamber. *p < 0.001 vs control; ^†^p < 0.002 vs LPS; ^‡^p < 0.001 vs LPS. n = 12 in each group.

VEGF significantly reduced TNF-α-induced albumin transfer to the lower chamber (*p *< 0.001; Fig. 3a). There was no significant difference in the albumin transfer between the control and the TNF-α + VEGF (each concentration) groups. VEGF (all concentrations) also significantly inhibited LPS-induced albumin transfer (*p *< 0.002, Fig. 3b). There was no significant difference in the albumin transfer between the control and the LPS + VEGF (500 ng/ml) groups, whereas the albumin transfer was significantly greater in the LPS + VEGF (100 ng/ml) group than in the control group (*p *< 0.001).

### TUNEL staining of HPAEC

**Figure 4:**
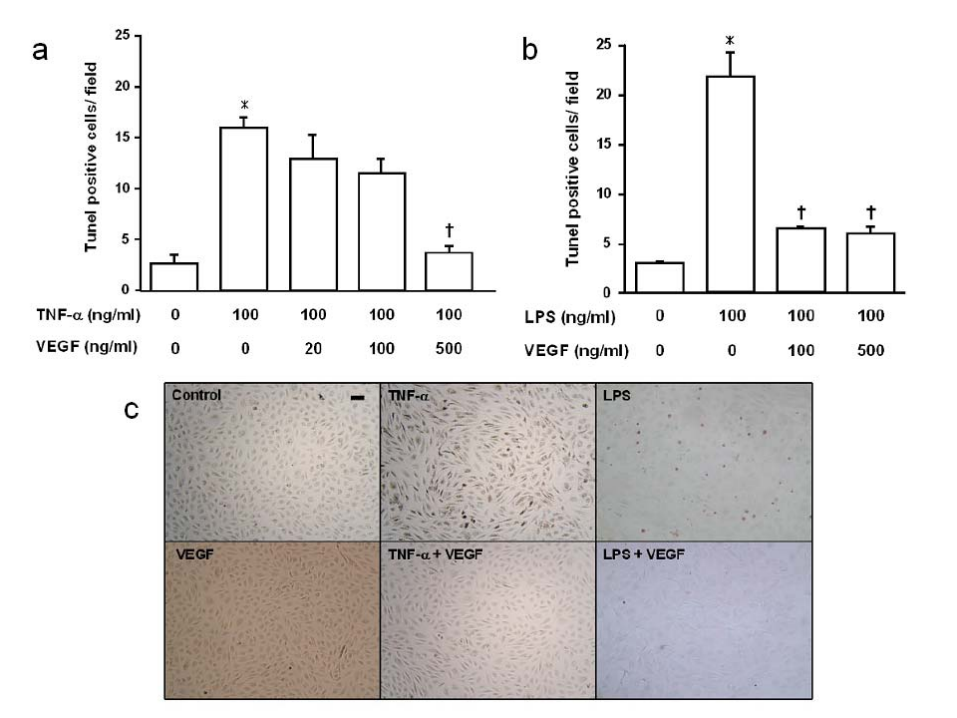
**Effect of VEGF on endothelial cell apoptosis assessed by TUNEL method**. **a) **The number of TUNEL positive cells after TNF-α stimulation for 24 h. *p < 0.001 vs control; †p < 0.001 vs TNF-α. **b) **The number of TUNEL positive cells after LPS stimulation for 24 h. *p < 0.0001 vs control; ^†^p < 0.001 vs LPS. n = 4 in each group. **c) **TUNEL method in HPAEC. Cells were unstimulated or stimulated by TNF-α, LPS, with or without the pretreatment of VEGF. Scale bar = 50 μm.

The TUNEL method was used to detect apoptotic cells. TNF-α treatment increased the number of apoptotic cells, which was significantly reduced by VEGF at 500 ng/ml (*p *< 0.001), but not at 100 ng/ml (Fig. 4a). LPS treatment also increased the number of apoptotic cells, and significantly fewer apoptotic cells were observed in the LPS+VEGF groups than in the LPS group (*p *< 0.001, Fig. 4b). There was no significant difference between control and LPS+VEGF groups. Representative photomicrographs of HPAEC after TUNEL staining are shown in Fig. 4c.

### Active caspase-3 immunoassay of HPAEC

**Figure 5:**
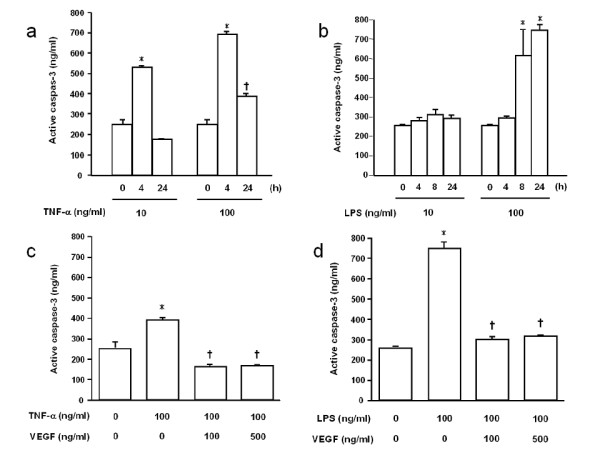
**Effect of VEGF on endothelial cell apoptosis assessed by active caspase-3 in cell lysates**. **a) **Stimulation with 100 ng/ml TNF-α stimulation for 4 and 24 h increased the levels of active caspase-3. *p < 0.0001 vs control; ^†^p < 0.01 vs control. **b) **LPS stimulation for 4, 8 and 24 h. Higher concentration (100 ng/ml) of LPS significantly increased active caspase-3 at 8 h and 24 h. *p < 0.0001 vs control. **c) **VEGF significantly reduced the levels of TNF-α-induced active caspase-3. *p < 0.0001 vs control; ^†^p < 0.0001 vs TNF-α. **d) **VEGF significantly decreased the levels of LPS-induced active caspase-3. *p < 0.0001 vs control; ^†^p < 0.0001 vs LPS. n = 4 in each group.

Active caspase-3 was detected with a human active caspase-3 immunoassay. Stimulation with TNF-α (10 ng/ml) for 4 h significantly increased the level of active caspase-3 compared with control (*p *< 0.0001), but, at 24 h, there was no significant difference between control and TNF-α-stimulated cells. Stimulation with 100 ng/ml TNF-α increased the levels of active caspase-3 compared with control at 4 h and 24 h (*p *< 0.0001 and *p *< 0.01, respectively, Fig. 5a). LPS (10 ng/ml) did not significantly increase the levels of active caspase-3. There was no significant difference between control and 10 ng/ml LPS at 4, 8 and 24 h, whereas 100 ng/ml LPS significantly increased active caspase-3 at 8 h and 24 h (*p *< 0.0001, Fig. 5b).

VEGF significantly reduced the levels of TNF-α-induced active caspase-3 (*p *< 0.001, Fig. 5c). There was no significant difference in active caspase-3 between the control and the TNF-α+VEGF groups. VEGF also significantly decreased the levels of LPS-induced active caspase-3 (*p *< 0.001, Fig. 5d). The levels of active caspase-3 in the control and the LPS+VEGF groups were similar. It was suggested that VEGF might suppress the activation of caspase-3 induced by TNF-α and LPS.

## Discussion

In the present study, we have observed that exogenous VEGF significantly decreased LPS-induced extravascular albumin leakage and edema formation. In contrast, VEGF blockade by monoclonal antibody enhanced tissue edema and neutrophil emigration on lung pathology. Pretreatment with VEGF significantly decreased the number of cells labeled with TUNEL staining and immunostaining of the pro-apoptotic proteins caspase-3, Bax, AIF, and cytochrome C. VEGF attenuated the increases in permeability of the HPAEC monolayer and the apoptosis of HPAECs induced by TNF-α and LPS. In addition, VEGF significantly reduced the levels of TNF-α- and LPS-induced active caspase-3 in HPAEC lysates. These results suggest that VEGF suppresses the apoptosis induced by inflammatory stimuli and plays a protective role during acute lung injury.

Apoptosis is an essential physiological process for the selective elimination of cells, which can be triggered by surface receptors, which interact with soluble proteins or membrane-bound proteins. Dysregulation of apoptosis pathways could contribute to the endothelial and epithelial injury that is characteristic of ALI/ARDS in humans [25]. In this study, pre-incubation with VEGF significantly attenuated the increase in the permeability of the endothelial monolayer, suggesting that VEGF protects against endothelial damage induced by TNF-α and LPS. To investigate the mechanism underlying this attenuation of endothelial damage, the effects of VEGF on apoptosis and activation of caspase-3, one of the effector caspases that initiate and execute cell death, were evaluated using HPAEC. Pre-incubation with VEGF significantly reduced the number of apoptotic cells and levels of active caspase-3, which is consistent with the observations of a previous study [19]. It was also reported that recombinant VEGF inhibited apoptosis of liver and renal cells and improved hepatic and renal dysfunctions during experimental pancreatitis [26]. They concluded that VEGF might function as a protective factor via the anti-apoptotic effect against the organ injury [26]. In this study, since TUNEL staining indicated attenuation of apoptosis of lung cells, we immunohistochemically evaluated the expression of four pro-apoptotic proteins, caspase-3, Bax, AIF and cytochrome C, in the lung. Bax is a member of the pro-apoptotic Bcl-2 family, which modulates death signaling and leads to the release of pro-apoptotic molecules from the mitochondrial intermembranous space, such as cytochrome C and AIF. Cytochrome C induces cell death by activation of caspase-9 and -3, whereas AIF leads to detrimental DNA damage by a caspase-independent pathway. We found that the expression of all of the apoptosis-associated molecules examined was attenuated by exogenous VEGF. Munshi and colleagues showed that VEGF inhibited the induction of Bax and activation of caspase-3 in LPS-induced endothelial apoptosis, a finding that is comparable to our result [27]. To the best of our knowledge, there has been no report on the effects of VEGF on the mitochondrial proteins AIF and cytochrome C. Pretreatment with VEGF inhibits the release of AIF and cytochrome C from the mitochondrial intermembranous space, although it remains to be determined which molecule of the apoptotic cascade VEGF affects. In addition to the inhibition of pro-apoptotic proteins, it was also reported that VEGF induces up-regulation of the anti-apoptotic proteins Bcl-2 and A1 in endothelial cells, which may be another mechanism for its inhibition of apoptosis [19,20]. Although other mechanisms could be involved, we concluded that VEGF-induced inhibition of apoptosis of endothelial cells might reduce albumin leakage through the pulmonary microvascular endothelium, leading to attenuation of lung injury.

In this study, we showed that VEGF attenuated the increases in the permeability of HPAEC monolayer and the apoptosis of HPAECs induced by TNF-α and LPS. It was also revealed that, during recovery from hyperoxic lung injury, when endothelial cells proliferate, VEGF mRNA becomes very abundant in alveolar type II cells [28]. In addition, the VEGF synthesized by the lung epithelial cells is known to contribute to the endothelial repair and angiogenesis processes following lung injury [29]. VEGF contributes to restoration of the cultured epithelial cell proliferation in an acid exposure model of injury [30]. We observed that the number of TUNEL-positive cells was decreased in the LPS+VEGF group compared with the LPS group. It is possible that VEGF may be associated with the protection and repair of the pulmonary endothelium and epithelium in the later phase of ALI/ARDS. Since our study limited its evaluation of the effects of VEGF to the acute phase of LPS-induced lung injury, the effects of exogenous VEGF or the anti-VEGF antibody at the later time points remain to be clarified.

In the lungs, VEGF mRNA is expressed primarily by alveolar epithelial cells and activated alveolar macrophages, which suggests that alveolar epithelial cells and macrophages may be potential sources of VEGF in the lungs [8,10,31]. VEGF protein is secreted by alveolar cell-like cell lines in response to a number of pro-inflammatory stimuli potentially involved in ALI/ARDS, such as LPS and neutrophil elastase [32]. However, a number of clinical investigations have shown that, during ALI/ARDS, the VEGF level in the lung was decreased, while the plasma concentration of VEGF is the same or increased [14-16,18,33]. The elevated plasma VEGF level could be derived from the molecule that constitutively exists in the alveolar space and leaks into the bloodstream [10]. In this study, we administered rmVEGF and anti-VEGF antibody intravenously. It is unclear whether rmVEGF and anti-VEGF antibody reached the alveolar space. However, it is not unreasonable to assume that they at least acted on the pulmonary vascular endothelial cells. Considering the impaired barrier function of vascular endothelium in the injured lung, we think it is possible that those agents administered intravenously might produce an effect on alveolar epithelial cells.

In this study, blockade of the VEGF cascade by anti-VEGF antibody significantly enhanced lung edema formation, as documented by the increased I/T ratio, after intratracheal LPS administration, although extravascular albumin leakage was not significantly changed. Usually, pathological changes occur after endothelial permeability is increased. We speculated that the difference in I/T ratio reached significance because the increase in the endothelial permeability was slight but sustained.

VEGF was originally discovered as a vascular permeability factor. Several *in vitro *studies revealed that VEGF treatment increases the endothelial permeability [4,34]. Kaner and coworkers delivered VEGF_165 _complementary DNA to the respiratory epithelium by an E1^- ^adenovirus vector and observed that overexpression of the VEGF gene induced pulmonary edema [13]. These previous observations indicate that VEGF increases endothelial permeability leading to pulmonary edema, whereas our data suggest that VEGF has a protective role during lung injury. Although it is difficult to compare the data from separate studies, we speculate that the function of VEGF may be different between normal lung and during severe lung inflammation. In *in vitro *and *ex vivo *studies using a variety of VEGF concentrations, the different species and methods used may have contributed to the apparent differences in the previously reported findings [35]. In a recent report, Mirzapoiazova and coworkers showed that the barrier properties of cultured HPAEC were improved by 10 ng/ml VEGF but decreased by 100 ng/ml VEGF [36]. They concluded that VEGF might cause diverse effects on pulmonary endothelial permeability depending on its concentration [36]. Another possible mechanism may be that the VEGF signaling pathway might be switched to an alternative pathway by the inflammatory stimuli or mediators that are associated with the pathogenesis of ALI/ARDS.

## Conclusion

In summary, the results of the present study suggest that VEGF may suppress the apoptosis cascade and attenuate the increased endothelial permeability and edema formation following intratracheal administration of inflammatory stimuli. We conclude that VEGF functions as a protective factor for the injured lung during the development of ALI/ARDS. Further investigation will be needed to determine whether VEGF or related molecules could be a therapeutic modality for this refractory disease.

## Competing interests

The author(s) declare that they have no competing interests.

## Authors' contributions

HK carried out animal studies and drafted the manuscript. ST conceived of the study, and participated in its design and coordination and helped to draft the manuscript. NH participated in the animal study. WY MS participated in the animal study. MN carried out the immunoassays. MY performed the permeability study using endothelial monolayers. EI carried out the immunoassays. YA participated in the design of the study and performed the statistical analysis. SF participated in in vitro measurements. KY participated in the animal study. AI conceived of the study, and participated in its design and coordination and helped to draft the manuscript. All authors read and approved the final manuscript.
